# Differential Exon Usage in Expression Profiles from Stress Axis Tissues of Brahman Cows Subjected to Prenatal Stress

**DOI:** 10.3390/genes17101208

**Published:** 2026-09-29

**Authors:** Kubra Z. Cilkiz, Emilie C. Baker, Charles R. Long, Ronald D. Randel, Noushin Ghaffari, Thomas H. Welsh, David G. Riley

**Affiliations:** 1 Department of Animal Science, Texas A&M University, College Station, TX 77843, USA; kubracilkiz@tarimorman.gov.tr (K.Z.C.); charles.long@ag.tamu.edu (C.R.L.); ronrandel1974@gmail.com (R.D.R.); 2 Department of Agricultural Sciences, West Texas A&M University, Canyon, TX 79016, USA; ebaker@wtamu.edu; 3 Texas A&M AgriLife Research & Extension Center at Overton, Overton, TX 75684, USA; 4 Department of Computer Science, Prairie View A&M University, Prairie View, TX 77446, USA; noghaffari@pvamu.edu; 5 Texas A&M AgriLife Research, College Station, TX 77843, USA

**Keywords:** differential exon usage, hypothalamic-pituitary-adrenal axis gene expression, prenatal transportation stress, Brahman cattle

## Abstract

Background/Objectives: Alteration in gene expression caused by stress may serve as a potential mechanism behind a range of differences previously observed in progeny exposed to prenatal stress. The objective of this study was to examine the impact of prenatal transportation stress on differential exon usage in expressed genes of stress-axis tissues of control and prenatally stressed Brahman cattle at five years of age. Methods: Pregnant Brahman cows were transported for 2 h sessions at 20-day intervals from the 60th to the 140th day of gestation, while pregnant herdmates served as controls. Blood samples were collected from six prenatally stressed (PNS) and eight control (control) females obtained when they were 5 years of age. Tissue samples were harvested from the adrenal medulla, adrenal cortex, anterior pituitary and hypothalamic paraventricular nucleus of the PNS and control cows at 5 years of age. Differential exon usage was analyzed by the DEXSeq tool to identify genes that were not previously identified by RNA sequencing analyses. Results: Differential exon usage (FDR ≤ 0.05) was detected in three exons (two genes) in the adrenal medulla, six exons (five genes) in the adrenal cortex, 13 exons (12 genes) in the anterior pituitary, and 37 exons (33 genes) in the hypothalamic paraventricular nucleus. Conclusions: The correspondence of prenatal stress to changes in exon usage across multiple tissues and genes could indicate investigative opportunities in postnatally affected physiological processes, including myelination, brain development, stress responses and metabolism.

## 1. Introduction

Alternative exon usage (i.e., differential exon usage) within genes is known to prominently occur in mammals [1]. Documentation of this differential usage might represent an opportunity for exploitation in livestock programs, especially with regard to response to stress. Trailer transportation of gestating cows represents an imposition of stress on the fetus [2] with many classic symptoms of stress, including variable concentrations of circulating cortisol, in both the dams and offspring [3,4,5]. Due to environmental pressure and selection on domesticated farm animals, they may be particularly vulnerable to detrimental effects of stress, as their metabolisms are oriented towards anabolism rather than defense systems, which are essentially catabolic [6]. Because energy is required to restore the animal’s bodily functions to homeostasis, stress increases the management requirements of animals and therefore reduces productivity and increases costs, leading to lower profitability [7,8,9]. Mechanisms opposing stress in neurobiological systems come from a series of complex pathways primarily involving the sympathomedullary system and the hypothalamic–pituitary–adrenal (HPA) axis [10,11]. The prenatal period is a critical time for development during which the brain and other organ systems are substantially formed. Thus, this is a period of vulnerability as the developing fetus is highly flexible and sensitive to environmental changes. Implicit in the developmental origin of diseases model is the concept that prenatal and early life exposure to a range of unfavorable experiences signals postnatal effects on physical and mental health [12].

Environmental or cellular stimuli cause changes in regulation of transcription, resulting in differences in the expression of genes. In addition, the differences in gene expression controlled by different regulatory mechanisms maintain the differentiation of all cells from each other. Differential exon usage and regulation have been reported in groups of genes related to fetal development in mice and humans [13,14].

Over half of genes exhibit distinct alternative spliced isoforms among different tissue types [15]. Differential exon usage analysis assesses differences across conditions between quantities that are directly observable from the RNA sequencing (RNA-seq) experiment’s reads, including relative exon usage. Differential exon usage is relevant because it may be involved in the production of different isoforms of proteins associated with normal or abnormal processes of development. In this study, we applied the statistical tool DEXSeq to identity and quantify prominent alterations in exon usage in specific genes in tissues of the stress response axis of mature cattle that experienced a prenatal stressor. We previously reported transcriptome analysis of the hypothalamic paraventricular nucleus, the anterior pituitary and the adrenal cortex using the same RNA-seq data previously to identify genes that were either downregulated or upregulated in 5-year-old cows that were exposed to prenatal transportation stress [16]. Evaluation of differential exon usage may reveal gene expression or activity differences that were not readily apparent in the RNA sequencing data. Differential exon usage has not been conducted in any HPA axis tissue of cattle. The hypothesis of this work was that assessing differential exon usage from genes within RNA sequencing results would identify active genes corresponding to prior, early life or prenatal exposure to stress in cattle. The objective of the present work was to augment that understanding based upon within-gene differential exon usage to (1) identify active HPA tissue genes in prenatally stressed cows by assessing differentially used exons and (2) reveal potentially altered metabolic or other pathways.

## 2. Materials and Methods

### 2.1. Animal Procedures

All animal experimental methods complied with the Guide for the *Care and Use of Agricultural Animals in Research and Teaching* [17] and were approved by the Texas A&M AgriLife Research Animal Care and Use Committee (Animal Use Protocol #2017-001A). All procedures were conducted in compliance with the *Guide for the Care and Use of Agricultural Animals in Research and Teaching*. The methodology and procedures used in this study were specifically detailed earlier [2,3,16,18]. In brief, the cows in the registered purebred Brahman herd at the Texas A&M AgriLife Research and Extension Center located in Overton, TX (32.27° N, −94.98° W) were artificially inseminated following naturally occurring estrus. At 45 d after the last breeding date, the stage of pregnancy was confirmed by palpation per rectum and compared with records of insemination dates. Pregnant cows were assigned randomly to the treatment (i.e., transportation) or control groups with respect to age (3 through 7 years), parity (1 through 4 calves previously born), and temperament evaluation (temperament score at weaning and mature temperament classification) in order to avoid confounding of these factors with the prenatal transportation treatment. Cows remained in their common pasture environment before and after a total of five transport events that occurred at 20 d intervals over 80 d of gestation (at 60, 80, 100, 120, and 140 ± 5 d of gestation). The treatment group, consisting of 48 cows, was transported in the same three-section trailer (2.4 m × 7.3 m) over the same smooth roadways and the same route for 2 h at an average of 75 km/h (150 km) in groups of 7 to 10 cows per transportation event. Low-stress handling techniques were followed to load cows on and off the trailer. Cows were always transported at the same time of the morning. Environmental temperature, which was recorded hourly for each date of transportation, was obtained from the research center’s weather station. The ambient temperature ranged from a high of 31.2 °C to a low of 14.1 °C over the course of this experiment. The control group of 48 pregnant cows was not subjected to transportation. Both the treatment and control groups of pregnant cows were managed together in the same pasture under identical nutritional conditions. The diet consisted of ad libitum coastal bermudagrass in pastures during the summer and fall. The same pastures were overseeded with rye (*Secale cereale*) and ryegrass (*Lolium multiflorum*) for winter grazing, with the cows supplemented with coastal bermudagrass (*Cynodon dactylon*) hay and a 3:1 corn:soybean meal mix as required depending on forage quality and availability.

From the transported cows, 20 male and 21 female calves (PNS) were born, while cows designated as the non-transported group gave birth to 26 male calves and 18 female calves (control). The 18 control and 21 PNS female calves were kept in the same pasture and nutritional conditions at the Texas A&M AgriLife Research and Extension Center in Overton until they were 5 years of age, when 8 nonpregnant, nonlactating cows from each group were designated at random for tissue collection. At 5 years of age, the stress axis tissues and peripheral blood leukocytes were collected from 8 control and 6 PNS cows. Eight cows per group were selected because a previous publication detected differences in bovine leukocyte DNA methylation patterns with seven individuals per treatment group [18]. Animal identification and treatment group designation were known to the investigators since the birth of the animals. However, tissues were collected from fewer PNS than control cows because two pregnant PNS cows were mistakenly transported to the cattle facility rather than the intended two nonpregnant PNS cows. This error was discovered when animal identification was confirmed before transport to the abattoir. The 5-year-old nonpregnant PNS (*n* = 6) and control (*n* = 8) Brahman cows were trailered to the departmental cattle facility and held for approximately 15 h in pens with free access to water before processing at the institution’s abattoir in the presence of a trained inspector from the Texas Department of Agriculture. The cows were harvested humanely between 0800 and 1100 h to obtain tissues of the stress axis. The cows were processed in a balanced alternating order (control, PNS, control, PNS, etc.) to minimize the potential confounders of time and order of processing. The abattoir staff members, trained in low-stress handling methods, quietly eased the cows into the stunning box. The staff promptly stunned the cows by using a captive bolt. Within 15 min of exsanguination, the stress axis tissues were promptly isolated, processed on site, stored in RNase/DNase-free sterile cryovials, snap-frozen in a portable liquid nitrogen tank, and then moved to the research laboratory in an adjacent building for storage at −80 °C.

### 2.2. Blood and Tissue Collection

The adrenal glands were removed and dissected free of adherent tissue within 15 min of slaughter. The adrenal cortex and adrenal medulla tissues were separated, and both tissues were placed in sterile cryovials, snap-frozen in liquid nitrogen, and stored at −80 °C to preserve RNA integrity. The anterior pituitary was carefully dissected free from the posterior pituitary and placed in liquid nitrogen after the intact pituitary gland was isolated from its surrounding tissues and connective membranes. A part of the brain encompassing the septum, preoptic region, and hypothalamus was removed to obtain tissue containing the hypothalamic paraventricular nucleus, instantly frozen on dry ice, before being stored at −80 °C until further processing. The frozen hypothalamic tissues were cut into 20 μm sections, which were then thaw-mounted on Superfrost/Plus glass microscope slides (Thermo Fisher Scientific, Waltham, MA, USA). After the position of the paraventricular nucleus was determined with Cresyl violet staining under bright-field microscopy, the frozen tissue was obtained from the slides by scraping and transferred to 500 μL of cold TRIzol Reagent (Thermo Fisher Scientific). The collected tissues were kept on ice for a few minutes and then stored at −80 °C. In the harvesting process, 10 mL of peripheral blood was acquired through a vacuum tube venipuncture to isolate leukocytes, as many studies use leukocytes as a surrogate for other tissues.

### 2.3. RNA Extraction and RNA Library Preparation

RNA was isolated from tissue samples that were cut to an average weight of 149 mg for the adrenal cortex, 132 mg for the adrenal medulla, and 158 mg for the anterior pituitary. The samples were then placed in 2 mL RNase-free sterile cryovials. For the paraventricular nucleus, 12 sections from each animal were utilized for RNA isolation to obtain at least 0.1 to 4 μg RNA. All samples were kept at −80 °C until they were analyzed. Blood samples went through centrifugation at 2671× *g* for 30 min at 6 °C. The white blood cell layers formed after centrifugation were transferred into 2 mL nuclease-free microcentrifuge tubes. The white blood cell layers were treated with a red blood cell lysis buffer solution repeatedly until a clear cell pellet was formed.

Frozen HPA axis tissues and leukocytes were processed by the Texas A&M Institute for Genome Sciences and Society (TIGSS) Experimental Genomics Core. After total RNA extraction was performed using the TRIzol Plus RNA Purification Kit (Thermo Fisher Scientific), quantification and quality assessment of purified RNA were conducted with the Qubit RNA Fluorometric Assay Kit (Thermo Fisher Scientific) and RNA ScreenTape Assay (Agilent Technologies, Santa Clara, CA, USA), respectively. Batch construction and sequencing lanes were randomized with respect to samples. The mean RIN was 7.3 across samples. Sequencing yielded an average of 49.63 million, 44.62 million, 28.3 million, and 24.22 million uniquely mapped reads for the adrenal cortex, adrenal medulla, pituitary gland, and paraventricular nucleus, respectively, before normalization.

Ribosomal RNA depletion was performed and an adapted globin approach was used for mRNA isolation from total RNA samples (TIGSS) of anterior pituitary, adrenal cortex, adrenal medulla and leukocytes using the TruSeq Stranded Total RNA Library Prep procedure with Ribo-Zero Globin (Illumina, Inc., San Diego, CA, USA). Processing and analysis of total RNA samples from the hypothalamic paraventricular nucleus were performed according to the high sample protocol of the Illumina TruSeq Stranded mRNA library preparation kit with a Dual Indexed RNA Adapter Plate (Illumina, Inc.). Because these protocols differ in transcript capture and detection sensitivity, direct comparisons of the number of differentially used exons across tissues should be interpreted with caution, as some portion of the observed differences may reflect differences in library preparation rather than tissue-specific biology. All libraries were sequenced at 150 bp paired end by the NovaSeq 6000 Sequencing System (Illumina, Inc.) after being verified through a 4200 TapeStation (Agilent Technologies) and qPCR.

### 2.4. RNA Library Processing and Alignment

**Figure 1:**
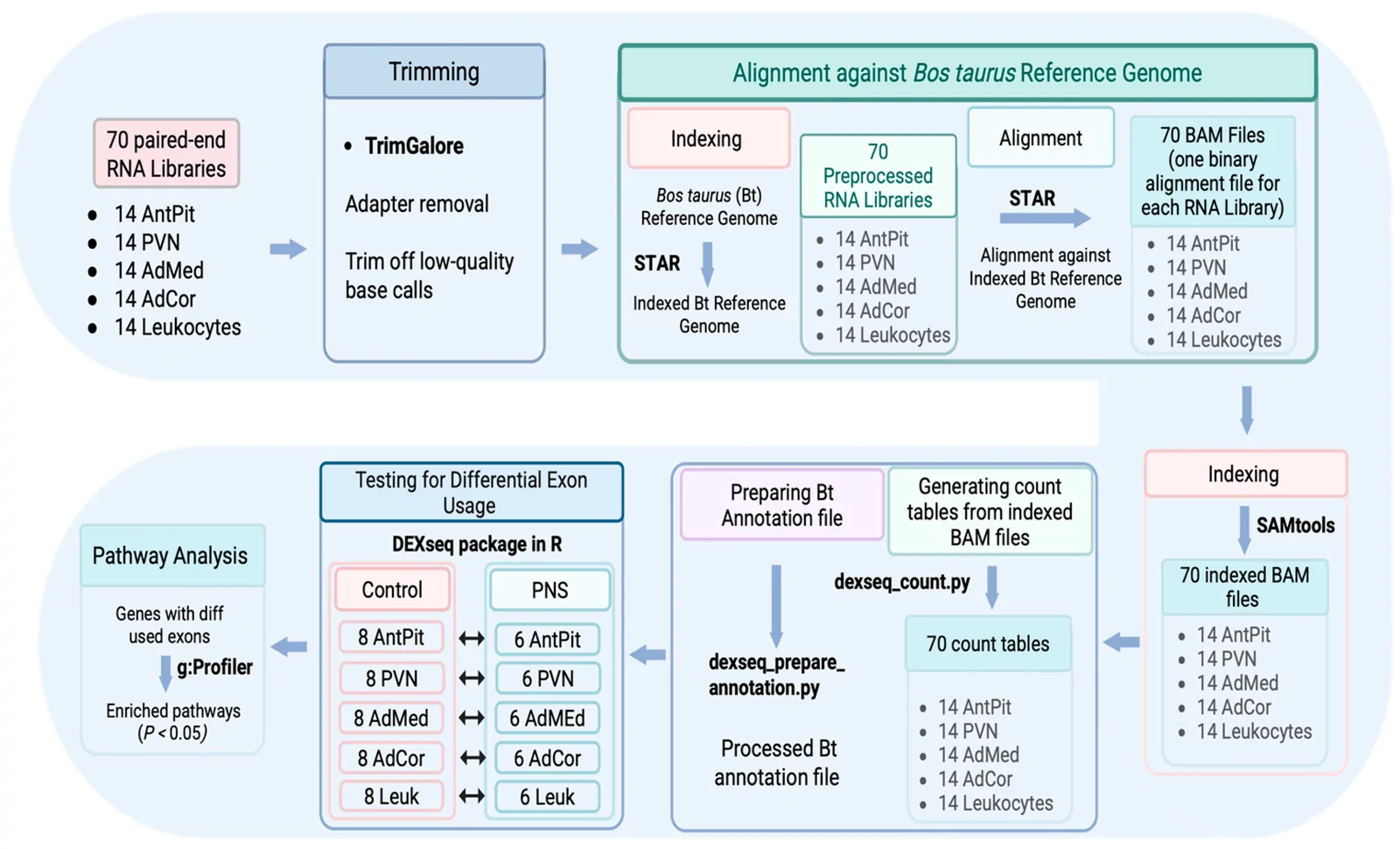
Workflow of differential usage of exons analysis. Software and scripts used in the associated step are depicted in bold: [TrimGalore version 0.6x, 19]; STAR version 2.4.0k [20]; dexseq_prepare_annotation.py; dexseq_count.py; DEXseq version 1.52.0 [22]; SAMtools version 1.14 [23];. Abbreviations: AntPit = anterior pituitary; PVN = hypothalamic paraventricular nucleus; AdMed = adrenal medulla; AdCor = adrenal cortex; Leuk = leukocytes.

The raw RNA FASTQ files were first processed with Trim Galore (version 0.6x) software (Babraham Bioinformatics, Cambridge, UK; [19]) to eliminate remaining adapter content and trim low-quality base calls from the 3′ ends of reads. Subsequently, the Spliced Transcripts Alignment to a Reference (STAR; version 2.4.0k) program [20] was utilized to generate an index file for the ARS-UCD1.2 genome assembly [21]. The trimmed reads were then aligned to the reference genome using the default STAR parameters, which have been specifically optimized for mammalian genome alignment. Low-expressed genes with less than 3 minimum reads per gene in at least 5 samples were removed. The analysis pipeline is depicted as a workflow in Figure 1.

### 2.5. Differential Exon Usage Analysis

The underlying principle of this analysis entails quantifying the reads that align to a particular exon and comparing this count with reads mapping to any other exons within the same gene. Observing changes in the ratio of these counts across conditions infers differences in relative exon usage. The software DEXSeq version 1.52.0 tool [22] was employed to statistically assess the observed differences in exon usage between treatments and the strength of these fluctuations through comparison of replicates before examining the averages between sample groups. Variance among the biological replicates was assessed assuming a negative binomial distribution, and deviances of generalized linear model fits were compared using a *χ*^2^ distribution. The design matrix of the statistical test was defined for animals and treatment. Dispersion was estimated with the generalized linear model (GLM) method. Estimates of dispersion were modeled with the negative binomial, and the contrast assessed was PNS minus the control group. Differential usage coefficients for exons were estimated as the logarithm of the fold change in bins corresponding to the exons relative to overall gene expression that represent the relative strength of the individual exons. Differentially utilized exons were declared with a false discovery rate (FDR) of 0.05 within tissue.

All binary alignment map files of aligned samples were indexed using SAMtools version 1.14 [23]. To prepare the annotation file (Bos_taurus.ARS-UCD1.2.98.gtf) for the differential exon usage analysis and generate a count table for each sample, publicly available Python scripts “dexseq_prepare_annotation.py” and “dexseq_count.py” were used, respectively. Within each tissue and leukocyte, 6 PNS samples and 8 control samples were compared and tested for differential exon usage using the DEXseq package [22] in R (R Core Team, 2021). Normalization factors were calculated using the trimmed mean of M-values (TMM) method. Genes identified with less than 3 minimum differentially used exons in at least 5 animals were not considered differentially used. Differential exon usage was assessed using a generalized linear model with terms for sample and exon identity, testing for a condition-by-exon interaction to determine whether relative exon usage differed between PNS and control groups (full model: ~sample + exon + condition:exon; reduced model: ~sample + exon). Differentially used exons were indicated by the number of the corresponding exon based on the gene annotations in the PNS group relative to the control group. Genes with identified differential exon usage, that is, only those that passed quality editing and met criteria for differential usage, from each tissue were used to investigate functional enrichment using Gene Ontology (GO) terms and pathway annotation using the KEGG and Reactome databases, implemented through the g:Profiler g:GOSt (https://biit.cs.ut.ee/gprofiler; accessed 16 September 2026) web server [24] with a significance threshold of FDR (Benjamini–Hochberg) < 0.05. The default annotated bovine genome was used for all tissues. All (*n* = 14) samples were included in tissue-specific analyses.

## 3. Results and Discussion

### 3.1. Differential Exon Usage Analysis

**Figure 2:**
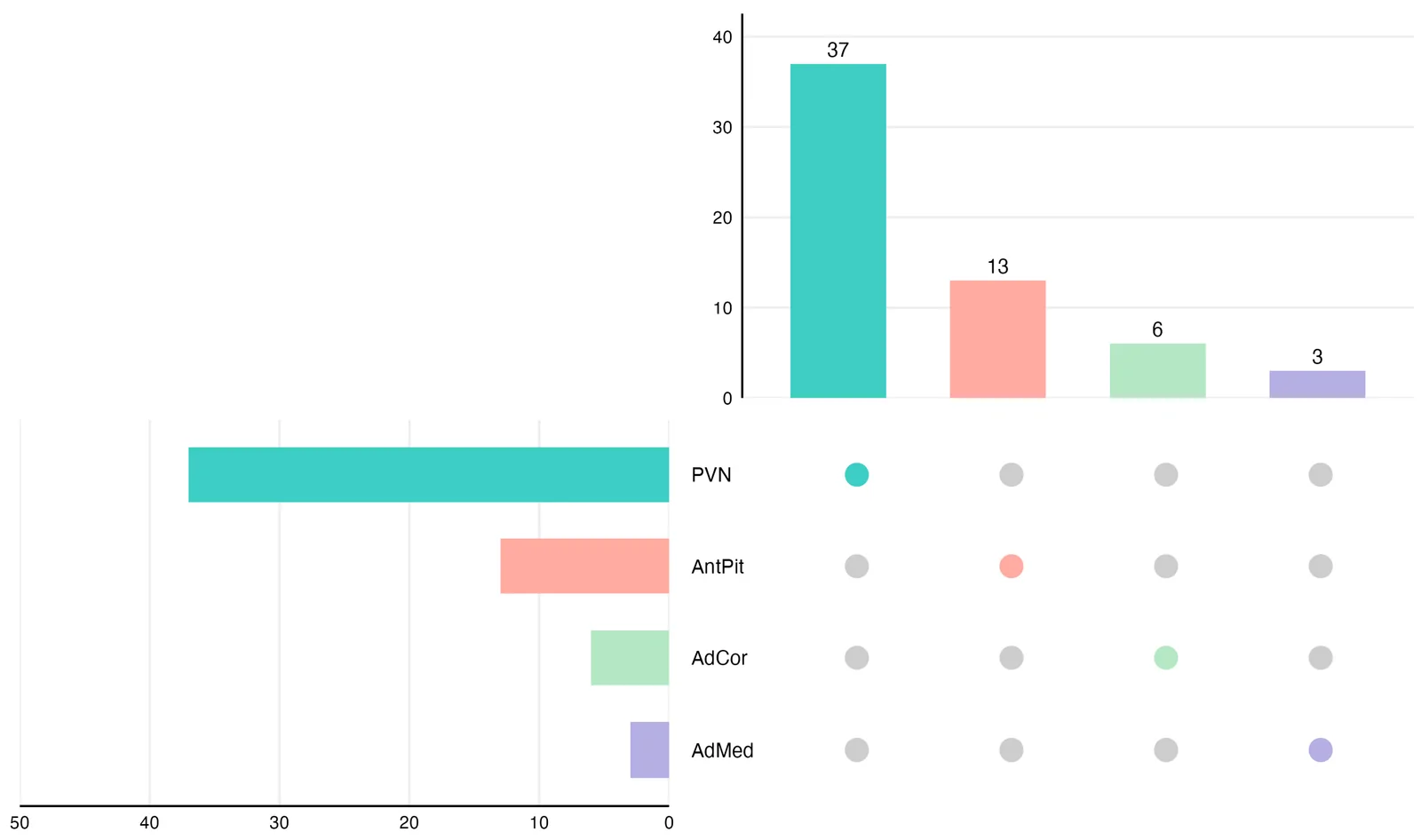
Number of differentially used exons according to the tissue type, with the corresponding number indicated above each bar. AntPit = anterior pituitary, PVN = hypothalamic paraventricular nucleus, AdMed = adrenal medulla, AdCor = adrenal cortex.

Multiple genes with differential exon usage were detected across tissue types in PNS compared to control. There were no differentially used exons detected in leukocytes. The number of differentially used exons across all tissue types is illustrated in Figure 2; notably, no differentially used exons within the same gene were identified across the different tissue types. As some genes harbored more than one differentially used exon, the number of genes was less than the number of exons, which will be discussed later. Of the genes with detected differentially used exons, those with previously confirmed roles in brain, neural, or stress response were of interest, although no concrete nor causative biological connection between the results of the present study and those genes should be inferred.

#### 3.1.1. Adrenal Medulla

There were three differentially used exons found in the adrenal medulla that met the significance threshold, with two belonging to the same gene (Table 1). The two most significant exons were the 18th and 19th exons in the actin-binding LIM protein 1 (*ABLIM1*) gene (FDR < 0.03). This gene attaches to the actin filaments and facilitates the interaction of actin filaments with targets in the cytoplasm using a LIM zinc-binding domain in its encoded protein [25] and functions downstream of DCC/netrin receptor signaling during axon guidance [26]. The *ABLIM1* gene also undergoes tissue-specific alternative splicing, with regulated inclusion of exons 11 and 12 producing distinct isoforms; loss of the exon 11-inclusion isoform in skeletal muscle has been linked to myotonic dystrophy type 1 (DM1) [27].

Exon 6 of the gene complement component 4A (*C4A*) was differentially used in the adrenal medulla (FDR < 0.03). Schizophrenia and autism spectrum disorder cases had higher levels of *C4A* expression in cortical regions of the brain [28], which might have relevance in brain tissue from stressed animals.

No statistically significant enrichment terms were identified for the adrenal medulla gene set that was identified to have differential exon usage; this likely reflects the small number of input genes.

#### 3.1.2. Adrenal Cortex

In the adrenal cortex, six exons in five distinct genes were identified as differentially used (FDR < 0.03) between PNS and control (Table 2). Differentially used exons 13 and 14 were in the DCC netrin 1 receptor (*DCC*) gene, which encodes a receptor that is a transmembrane protein which guides axons of neurons by facilitating their cones growing towards netrin 1 ligand sources. This gene is highly expressed by dopaminergic neurons. Changes in the expression level of the *Dcc* gene early in life might be associated with the development of dopaminergic dysregulation found in certain psychiatric conditions like schizophrenia or as a result of prolonged drug use in *Dcc*-deficient mice [29], and others also noted a connection between adolescent mouse deficiencies in *Dcc* expression and adult neurological conditions [30]. One of the most significant exons was the part of the amyloid beta precursor protein-binding family B member 2 (*APBB2*; FDR < 0.03). This gene is involved in the branching of neurons in the hippocampus and the development of neuromuscular junctions. It was differentially methylated and proposed as one of the mediators between child cognitive ability and cumulative prenatal risk factors like substance use and physical and mental health [31] and earlier identified as integral and influential in cognitive impairment accumulated by aging [32]. Platelet endothelial aggregation receptor-1 (*PEAR1*) has documented expression in *Homo sapiens* brain and neural tissue [33], and phosphofurin acidic cluster sorting protein 2 (*PACS2)* is an instigator in neurological development, and its disruptions are profound [34,35]. Speculatively, these genes might have a role in mediating an animal’s response to physical or psychogenic stressors.

**Figure 3:**
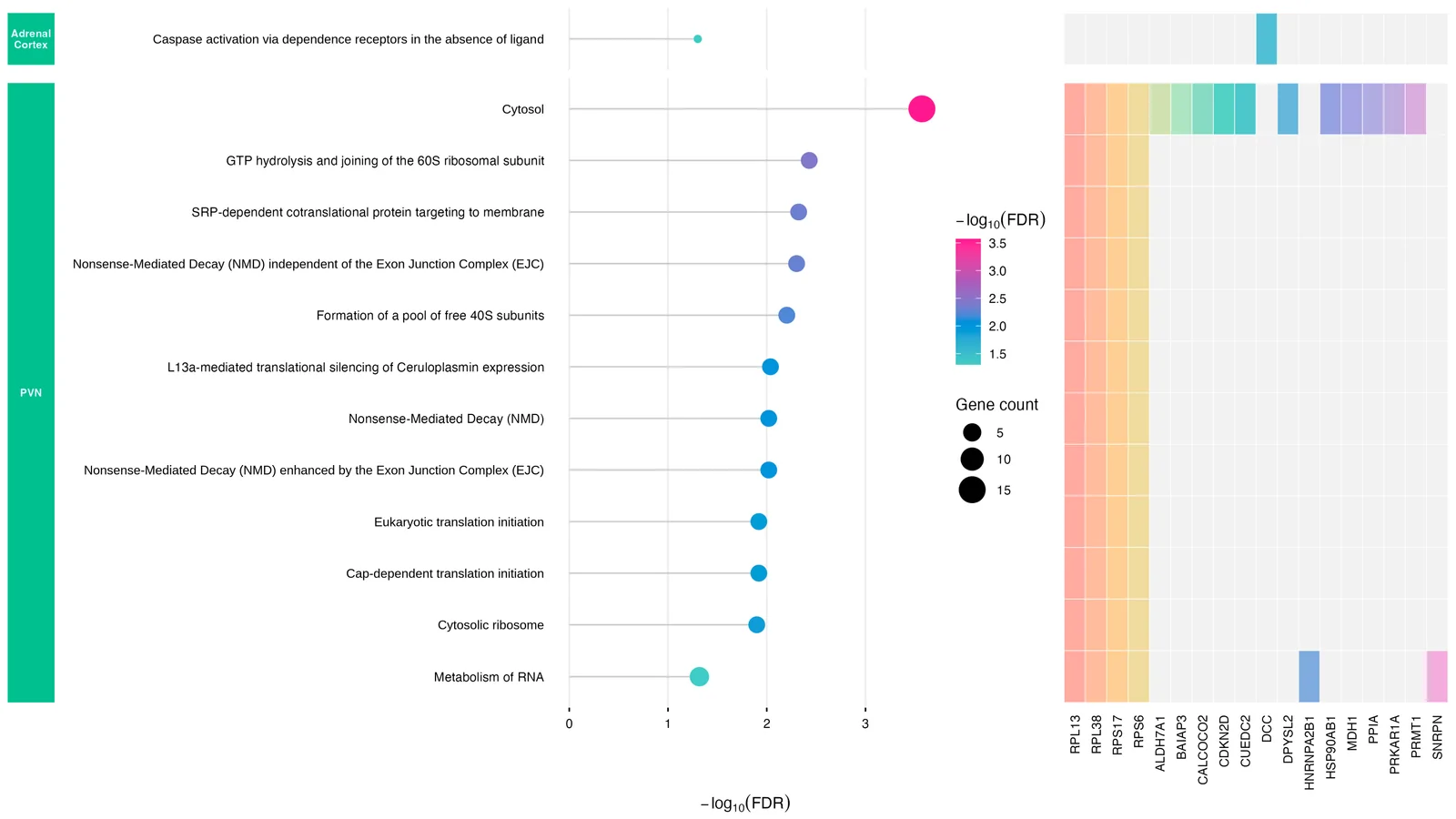
Tissue-specific functional enrichment profiling of genes with differential exon usage. The left panel shows enriched terms from KEGG, Gene Ontology, and Reactome (REAC) databases, grouped by tissue. Dot position and color indicate statistical significance (−log_10_ FDR), and dot size indicates the number of genes contributing to each enrichment. The right panel shows the presence and absence of all 18 enriched genes by tissue. PVN = hypothalamic paraventricular nucleus.

A single Reactome pathway was significantly enriched in the adrenal cortex (Figure 3; Supplementary Table S1), with the Reactome result corresponding to the DCC gene discussed above. This finding might suggest the potential role of differential exon usage in modulating apoptotic signaling within adrenocortical tissue.

#### 3.1.3. Anterior Pituitary

There were 13 exons in 12 genes in the anterior pituitary that were differentially used (FDR < 0.05; Table 3). Many of the genes have been documented with some expression in the brain or nervous system in other species. Of special interest is the differential exon usage in ornithine decarboxylase 1 (*ODC1*), because of its activity during embryonic development, especially from implantation to the fetal stage [35,36,37]. Dihydropyrimidinase-like 5 (*DPYSL5*) variants have been confirmed as causal for brain disorders and neurodevelopmental perturbation [38,39,40]. Expression of this gene was downregulated in the anterior pituitary of the same PNS cows [16]. Another gene with a differentially used exon in the anterior pituitary with embryogenic developmental impacts, especially with respect to body plan execution, is striatin-interacting protein 1 (*STRIP1*; [41,42]). V-Set and transmembrane domain-containing 4 (*VSTM4*) have a regulatory role with T cells [43], and its expression in the placenta and brain was altered by a prenatal stressor of pesticide exposure [44]. Ubiquitin-specific peptidase 40 (*USP40*) and armadillo repeat-containing 5 (*ARMC5*) are documented with expression in brain tissue and correspond to aging-related neurological disorders [45,46]. CCR4-NOT transcription complex subunit 1 (*CNOT1*) and Rho GTPase-activating protein 18 (*ARHGAP18*) have high *H. sapiens* brain expression and were implicated in abnormal brain and neural cell development [47,48]. This gene has also been implicated in schizophrenia [49] and, when activated by ezrin, inactivates RhoA to regulate actin cytoskeleton organization [50]. Since RhoA-dependent actin remodeling is known to regulate hormone secretion in neuroendocrine cells [51], differential exon usage in *ARHGAP18* may have an undescribed role for secretion of hormones synthesized by the various cell types of the anterior pituitary gland, as well.

No significant enrichment terms were identified.

#### 3.1.4. Hypothalamic Paraventricular Nucleus

There were 37 exons that were differentially used (FDR < 0.05) in 33 genes between PNS and control in the hypothalamic paraventricular nucleus, as detailed in Table 4. The neuropeptide C-type natriuretic peptide (*CNP*) is linked to modulation of blood pressure and fluid balance, two physiological parameters relevant to stressors, such as transportation. In addition, CNP supports maintenance of the blood–brain barrier, the integrity of which is vital during exposure to pathogenic stressors. These are homeostatic functions of relevance when an animal’s health and survival depend on successfully responding to stressful situations [52,53]. Seven of the 33 noted genes encode ribosomal proteins; two and three exons had differential usage in *RPS6* and *RPS17.* Three genes are involved in splicing or its regulation, including transmembrane BAX inhibitor motif-containing 6 (*TMBIM6*), CDC-like kinase 1 (*CLK1*), and serine and arginine-rich splicing factor 4 (*SRSF4*) [54,55,56,57]. This suggests another regulatory role important in bovine response to stress. Three exons in peptidylprolyl isomerase A (*PPIA*) and two in ubiquitin-specific peptidase 11 (*USP11*) were differentially used; neither gene has a noted role in brain or neural tissues.

Many of the genes with exons differentially used in the hypothalamic paraventricular nucleus are influential in brain or nervous system maladies or disorders in *H. sapiens.* These included aldehyde dehydrogenase 7 family member A1 (*ALDH7A1*; [58]), heat shock protein 90 alpha family class B member 1 (*HSP90AB1*; [59]), malate dehydrogenase 1 (*MDH1*; [60,61]), dihydropyrimidinase-like 2 (*DPYSL2*; [39]), arginine-glutamic acid dipeptide repeats (*RERE*; [62,63,64], CUE domain-containing 2 (*CUEDC2*; [65]), progestin and adipoQ receptor family member 6 (*PAQR6*; [66]), and BAI1-associated protein 3 (*BAIAP3*; [67]). A group of genes with differential exon usage are involved in neuronal or other aspects of nervous system development. These include SPRY domain-containing 3 (*SPRYD3*; [68]), *DPYSL2* [40], cyclin-dependent kinase inhibitor 2D (*CDKN2D*; [69]), calcium-binding and coiled-coil domain 2 (*CALCOCO2*; [70]), and mal, T cell differentiation protein (*MAL*; [71]). These possibly could influence stress response in animals.

The genes with differentially used exons were almost completely distinct from differentially expressed genes in the same tissues of the same cows [16], with the lone exception being the downregulation of *DPYSL5* in the anterior pituitary. Many more active genes were identified in the present study in the hypothalamic paraventricular nucleus and the anterior pituitary than in the prior RNA-seq work [16]. This difference in abundance of identified genes may reflect the necessity of the hypothalamic paraventricular nucleus to rapidly integrate intrinsic and extrinsic signals to appropriately orchestrate the response of the anterior pituitary gland and the adrenal gland to a stressor.

Enrichment analysis of the hypothalamic paraventricular nucleus gene set that was identified with differential exon usage revealed a strong signal related to ribosomal and translational machinery, with the genes *RPS6*, *RPL38*, *RPS17*, and *RPL13* collectively driving eight of the significantly enriched Reactome terms, including pathways for ribosomal subunit assembly, cap-dependent translation initiation, and nonsense-mediated decay (Figure 3). This same gene set also underlies the enriched GO cellular component terms “cytosol” and “cytosolic ribosome.” This aligns with reports that glucocorticoid signaling remodels protein synthesis pathways, including a dexamethasone-induced enrichment for GTP hydrolysis and ribosome biogenesis genes in human PSC-derived neural models [72]. Because ribosomal protein mRNAs are subject to tightly regulated translational control in response to hormonal and stress-related cues [73] and splicing of ribosomal protein genes has itself been shown to be stress-responsive in other systems [74], the differential exon usage of *RPS6*, *RPL38*, *RPS17*, and *RPL13* observed here between prenatally stressed and control cattle may reflect a lasting, glucocorticoid-mediated reprogramming of ribosome biogenesis within the PVN. Together, these findings may point to a link between glucocorticoid signaling and ribosome biogenesis within the PVN. However, this interpretation should be approached cautiously, as the enriched pathways are supported by a relatively small, overlapping set of genes, and direct assessment of glucocorticoid receptor activity or ribosome output would be needed to confirm this relationship.

*RPS6*, *RPL38*, *RPS17*, and *RPL13* are core structural components of the ribosome, and it was differential exon usage in these four genes alone that drove enrichment across ribosomal subunit assembly, cap-dependent translation initiation, and nonsense-mediated decay. The repeated occurrence of these genes is biologically notable, as changes in exon usage within ribosomal protein genes may have meaningful functional consequences. Altered exon usage in ribosomal protein genes may have functional consequences, as alternative splicing can generate distinct ribosomal protein isoforms that differ in their incorporation into or association with assembled ribosomes [75]. Such changes may contribute to ribosome heterogeneity and, in some cases, influence translational activity or mRNA selectivity [76]. Whether the exon usage differences observed here in *RPS6*, *RPL38*, *RPS17*, and *RPL13* correspond to altered ribosomal protein isoforms or ribosome composition in the PVN has not been established in this study and would require direct validation.

Caveats in interpretation of these results are common to many gene expression studies. A limited sample size may not adequately reflect reality. RNA sequencing studies that could confirm relevant exon-containing transcripts, particularly for high-priority genes such as *DCC*, *ABLIM1*, or the ribosomal/splicing-related genes identified in the hypothalamic paraventricular nucleus would be desirable and valuable independent validation efforts. Results may not characterize in all cattle breeds the consequences of other types of prenatal stressors, such as external insults or nutrient limitations.

## 4. Conclusions

The 5 yr old mature PNS cows used in this study developed in utero in a prenatal environment that was physiologically different from control cows. The mothers of the PNS group experienced increased vaginal temperature and serum concentrations of cortisol and glucose in response to the transportation events during gestation [3]. Post-parturition, as suckling calves, the PNS group was more temperamental and had increased serum cortisol relative to the control group [5]. This early-in-life difference in serum cortisol was also evident at maturity in the control (29.5 ± 9.8 ng/mL) and PNS (40.34 ± *5.2* ng/mL) cows [77]. Differential exon usage analysis of stress axis tissues from mature Brahman cows that were prenatally stressed relative to non-stressed mature control cows resulted in the identification of genes that were not previously identified in gene expression analyses of sequenced RNA populations of the hypothalamic paraventricular nucleus, the anterior pituitary or the adrenal cortex [16]. There were no differentially used exons detected in leukocytes. Genes with differentially used exons in the adrenal cortex included *DCC*, *PEAR1*, *PACS2*, and *APBB2*. Genes related to brain or neural processes that had differentially used exons in the anterior pituitary included *ARMC5*, *CNOT1*, and *ARHGAP18*. Also in the anterior pituitary, genes with differentially used exons included some with prominent activity during embryonic development, including *DPYSL5*, *STRIP1,* and *VSTM4*. Genes in the hypothalamic paraventricular nucleus with differentially used exons included several encoding ribosomal proteins and three involved in splicing or its regulation, as well as a neuropeptide (*CNP*) involved in fluid balance and vascular tone. The results reported herein provide evidence of in utero exposure to a stressor (i.e., prenatal transportation stress) as an association between prenatal transportation stress and differential exon usage detected at five years of age. The demonstrated association of differential exon usage within genes with prenatal stress should be investigated for possible effects on cattle health and productivity through the neural function, metabolism, and immune pathways identified. Additional efforts to characterize differential exon usage in the presence of stressors need to be approached at a younger age, that is, closer to the occurrence of the stressor.

## Figures and Tables

**Table 1:** Exon usage coefficients for differentially used exons in the adrenal medulla ^1^.

Gene	BTA ^2^	Exon ^3^	bp ^4^	Control ^5^	PNS ^5^	FDR ^6^
Actin-binding LIM protein 1 (*ABLIM1)*	26	19	35,007,753	11.7	14.3	0.004
		18	35,005,719	12.2	14.9	0.025
Complement component 4A (*C4A)*	23	6	27,373,723	14.6	10.5	0.025

^1^ Differential usage coefficients for exons were estimated as the logarithm of the fold change in bins corresponding to the exons relative to overall gene expression that represent the relative strength of the individual exons. ^2^ *Bos taurus* autosome. ^3^ The exon number within the gene. ^4^ Start (centromeric) exon boundary. ^5^ Treatment groups: control and prenatally stressed (PNS). ^6^ False discovery rate: FDR.

**Table 2:** Exon usage coefficients for differentially used exons in the adrenal cortex ^1^.

Gene	BTA ^2^	Exon ^3^	bp ^4^	Control ^5^	PNS ^5^	FDR ^6^
DCC Netrin 1 Receptor (*DCC*)	24	14	52,806,532	5.8	1.9	<0.001
		13	52,779,607	5.3	2.4	0.032
Platelet endothelial aggregation receptor 1 (*PEAR1*)	3	15	13,945,979	5.4	8.3	0.003
Phosphofurin acidic cluster sorting protein 2 (*PACS2*)	21	25	69,682,467	27.6	25.6	0.003
LOC107131209	23	25	27,342,714	18.0	28.4	0.022
Amyloid beta precursor protein-binding family B member 2 (*APBB2*)	6	23	60,095,654	7.4	10.1	0.022

^1^ Differential usage coefficients for exons were estimated as the logarithm of the fold change in bins corresponding to the exons relative to overall gene expression that represent the relative strength of the individual exons. ^2^ *Bos taurus* autosome. ^3^ The exon number within the gene; larger numbers indicate more distal, from the acrocentric centromere, coordinates. ^4^ Start (centromeric) exon boundary. ^5^ Treatment groups: control and prenatally stressed (PNS). ^6^ False discovery rate: FDR.

**Table 3:** Exon usage coefficients for differentially used exons in the anterior pituitary ^1^.

Gene	BTA ^2^	Exon ^3^	bp ^4^	Control ^5^	PNS ^5^	FDR ^6^
S100 calcium-binding protein A14 (*S100A14*)	3	13	16,767,853	22.9	24.7	0.002
Splicing factor 3a subunit 2 (*SF3A2*)	7	15	21,403,165	7.6	4.8	0.003
LDL receptor-related protein 1 (*LRP11*)	9	4	86,985,452	11.3	9.6	0.003
Enah/vasp-like (*EVL*)	21	4	65,004,289	1.5	4.3	0.014
CCR4-NOT transcription complex subunit 1 (*CNOT1*)	18	42	26,351,832	5.8	2.9	0.014
Cytochrome P450 family 4 subfamily V member 2 (*CYP4V2*)	27	7 6	16,257,456 16,256,121	11.0 9.5	9.4 7.8	0.014 0.04
Armadillo repeat-containing 5 (*ARMC5*)	25	1	27,493,317	5.6	3.4	0.04
Ornithine decarboxylase 1 (*ODC1*)	11	3	87,200,438	13.0	10.9	0.04
V-set and transmembrane domain-containing 4 (*VSTM4*)	28	1	43,285,950	14.0	15.5	0.04
Rho GTPase-activating protein 18 (*ARHGAP18*)	9	15	67,796,361	11.4	9.8	0.04
Dihydropyrimidinase-like 5 (*DPYSL5*)	11	7	72,701,213	6.8	4.5	0.04
Striatin-interacting protein 1 (*STRIP1*)	3	22	33,389,790	7.1	4.9	0.046

^1^ Differential usage coefficients for exons were estimated as the logarithm of the fold change in bins corresponding to the exons relative to overall gene expression that represent the relative strength of the individual exons. ^2^ *Bos taurus* autosome. ^3^ The exon number within the gene; larger numbers indicate more distal, from the acrocentric centromere, coordinates. ^4^ Start (centromeric) exon boundary. ^5^ Treatment groups: control and prenatally stressed (PNS). ^6^ False discovery rate: FDR.

**Table 4:** Exon usage coefficients for differentially used exons in the hypothalamic paraventricular nucleus ^1^.

Gene	BTA ^2^	Exon ^3^	bp ^4^	Control ^5^	PNS ^5^	FDR ^6^
Heterogeneous nuclear ribonucleoprotein A2B1 (*HNRNPA2B1*)	4	15	69,743,057	37.1	39.3	0.002
Ubiquitin-specific peptidase 11 (*USP11*)	X	18	85,617,408	24.2	26.2	0.002
C-type natriuretic peptide (*CNP*)	19	1	42,130,921	24.7	22.4	0.002
Ribosomal protein S6 (*RPS6*)	8	1	24,916,124	30.1	27.8	0.004
Ribosomal protein S27a (*RPS27A*)	11	8	37,972,454	40.3	41.5	0.012
Ribosomal protein L38 (*RPL38*)	19	1	57,192,444	25.4	26.9	0.012
CDC-like kinase 1 (*CLK1*)	2	11	89,508,984	29.1	27.5	0.016
Peptidylprolyl isomerase A (*PPIA*)	4	4	76,710,886	43.8	42.1	0.016
Transmembrane BAX inhibitor motif-containing 6 (*TMBIM6*)	5	5	30,116,464	33.2	32.2	0.016
SPRY domain-containing 3 (*SPRYD3*)	5	9	26,900,923	25.6	24.3	0.016
Progestin and adipoQ receptor family member 6 (*PAQR6*)	3	4	14,547,753	25.5	23.5	0.016
Uncharacterized gene	23	10	28,665,069	23.9	20.6	0.018
Ribosomal protein S17 (*RPS17*)	21	1	22,839,036	36.5	38.4	0.02
C9H6orf120	9	2	103,493,084	23.0	22.9	0.02
Serine and arginine-rich splicing factor 4 (*SRSF4*)	2	6	124,392,151	21.9	20.3	0.022
CUE domain-containing 2 (*CUEDC2*)	26	1	23,046,322	23.8	25.9	0.022
Cilia and flagella-associated protein 36 (*CFAP36*)	11	7	38,237,418	21.9	20.3	0.022
*RPS17*	21	4	22,842,018	35.3	36.4	0.024
Cyclin-dependent kinase inhibitor 2D (*CDKN2D*)	7	2	15,071,373	48.7	47.8	0.024
Arginine-glutamic acid dipeptide repeats (*RERE*)	16	26	45,024,992	30.0	28.6	0.024
Dihydropyrimidinase-like 2 (*DPYSL2*)	8	19	73,873,202	20.4	22.1	0.024
*RPS6*	8	4	24,917,750	44.6	45.6	0.024
Small nuclear ribonucleoprotein polypeptide N (*SNRPN*)	21	10	1,956,204	41.5	40.7	0.026
*PPIA*	4	1	76,709,520	52.9	54.9	0.028
		5	76,713,151	41.9	39.8	0.028
Ribosomal protein L13 (*RPL13*)	18	7	14,492,410	25.2	27.8	0.031
Protein arginine methyltransferase 1 (*PRMT1*)	18	7	56,083,623	25.2	23.8	0.031
Malate dehydrogenase 1 (*MDH1*)	11	8	62,045,389	44.1	43.5	0.032
BAI1-associated protein 3 (*BAIAP3*)	25	11	1,063,493	30.5	28.5	0.039
Proteasome activator subunit 1 (*PSME1*)	10	14	21,014,236	22.7	21.3	0.039
Aldehyde dehydrogenase 7 family member A1 (*ALDH7A1*)	7	19	27,317,378	31.7	34.7	0.04
Calcium-binding and coiled-coil domain 2 (*CALCOCO2*)	19	8	37,687,408	23.1	21.6	0.041
Heat shock protein 90 alpha family class B member 1 (*HSP90AB1*)	23	9	17,756,566	44.3	43.6	0.041
Protein kinase CAMP-dependent type I regulatory subunit alpha (*PRKAR1A*)	19	3	61,705,135	26.1	28.1	0.041
Ubiquitin-specific peptidase 11 (*USP11*	X	15	85,616,397	23.5	22.0	0.042
Mal, T cell differentiation protein (MAL blood group) (*MAL*)	11	2	1,848,722	23.9	25.2	0.047
*RPS17*	21	2	22,840,459	41.8	43.0	0.049

^1^ Differential usage coefficients for exons were estimated as the logarithm of the fold change in bins corresponding to the exons relative to overall gene expression that represent the relative strength of the individual exons. ^2^ *Bos taurus* autosome. ^3^ The exon number within the gene; larger numbers indicate more distal, from the acrocentric centromere, coordinates. ^4^ Start (centromeric) exon boundary. ^5^ Treatment groups: control and prenatally stressed (PNS). ^6^ False discovery rate: FDR.

## Data Availability

Data will be made available upon reasonable request.

## Supplementary Materials

The following supporting information can be downloaded at: https://www.mdpi.com/article/10.3390/genes17101208/s1, Supplementary Table S1: Functional enrichment analysis of genes with differentially used exons by tissue.

## References

[1] Barbosa-Morais N.L., Irimia M., Pan Q., Xiong H.Y., Gueroussov S., Lee L.J., Slobodeniuc V., Kutter C., Watt S., Çolak R. (2012). The Evolutionary Landscape of Alternative Splicing in Vertebrate Species. Science.

[2] Lay D.C., Friend T.H., Randel R.D., Jenkins O.C., Neuendorff D.A., Kapp G.M., Bushong D.M. (1996). Adrenocorticotropic Hormone Dose Response and Some Physiological Effects of Transportation on Pregnant Brahman Cattle. J. Anim. Sci..

[3] Price D.M., Lewis A.W., Neuendorff D.A., Carroll J.A., Burdick Sanchez N.C., Vann R.C., Welsh T.H., Randel R.D. (2015). Physiological and Metabolic Responses of Gestating Brahman Cows to Repeated Transportation. J. Anim. Sci..

[4] Lay D.C., Randel R.D., Friend T.H., Jenkins O.C., Neuendorff D.A., Bushong D.M., Lanier E.K., Bjorge M.K. (1997). Effects of Prenatal Stress on Suckling Calves. J. Anim. Sci..

[5] Littlejohn B.P., Price D.M., Banta J.P., Lewis A.W., Neuendorff D.A., Carroll J.A., Vann R.C., Welsh T.H., Randel R.D. (2016). Prenatal Transportation Stress Alters Temperament and Serum Cortisol Concentrations in Suckling Brahman Calves. J. Anim. Sci..

[6] Judge M.D., Briskey E.J., Meyer R.K. (1966). Endocrine Related Post-Mortem Changes in Porcine Muscle. Nature.

[7] Carroll J.A., Forsberg N.E. (2007). Influence of Stress and Nutrition on Cattle Immunity. Vet. Clin. N. Am. Food Anim. Pract..

[8] Cahill S., Chandola T., Hager R. (2022). Genetic Variants Associated with Resilience in Human and Animal Studies. Front. Psychiatry.

[9] Richeson J.T., Weigand M.C., Samuelson K.L., Falkner R. (2026). Practical Considerations of Resilience and Robustness Within the Stress Architecture of Beef Stocker Calves. Vet. Clin. N. Am. Food Anim. Pract..

[10] Minton J.E. (1994). Function of the Hypothalamic-Pituitary-Adrenal Axis and the Sympathetic Nervous System in Models of Acute Stress in Domestic Farm Animals. J. Anim. Sci..

[11] Smith S.M., Vale W.W. (2006). The Role of the Hypothalamic-Pituitary-Adrenal Axis in Neuroendocrine Responses to Stress. Dialogues Clin. Neurosci..

[12] Gluckman P.D., Hanson M.A. (2004). Developmental Origins of Disease Paradigm: A Mechanistic and Evolutionary Perspective. Pediatr. Res..

[13] Denkena J., Zaisser A., Merz B., Klinger B., Kuhl D., Blüthgen N., Hermey G. (2020). Neuronal Activity Regulates Alternative Exon Usage. Mol. Brain.

[14] Do H.T.T., Shanak S., Barghash A., Helms V. (2023). Differential Exon Usage of Developmental Genes Is Associated with Deregulated Epigenetic Marks. Sci. Rep..

[15] Liu Q., Fang L., Wu C. (2022). Alternative Splicing and Isoforms: From Mechanisms to Diseases. Genes.

[16] Earnhardt-San A.L., Baker E.C., Cilkiz K.Z., Cardoso R.C., Ghaffari N., Long C.R., Riggs P.K., Randel R.D., Riley D.G., Welsh T.H. (2025). Evaluation of Prenatal Transportation Stress on DNA Methylation (DNAm) and Gene Expression in the Hypothalamic–Pituitary–Adrenal (HPA) Axis Tissues of Mature Brahman Cows. Genes.

[17] McGlone J., Ford S., Mitloehner F., Grandin T., Ruegg P., Stull C., Lewis G., Swanson J., Underwood W., Mench J. (2010). Guide for the Care and Use of Agricultural Animals in Research and Teaching.

[18] Littlejohn B.P., Price D.M., Neuendorff D.A., Carroll J.A., Vann R.C., Riggs P.K., Riley D.G., Long C.R., Welsh T.H., Randel R.D. (2018). Prenatal Transportation Stress Alters Genome-Wide DNA Methylation in Suckling Brahman Bull Calves. J. Anim. Sci..

[19] Krueger F. (2023). FelixKrueger/TrimGalore: V0.6.10—Add Default Decompression Path. Zenodo.

[20] Dobin A., Davis C.A., Schlesinger F., Drenkow J., Zaleski C., Jha S., Batut P., Chaisson M., Gingeras T.R. (2013). STAR: Ultrafast Universal RNA-Seq Aligner. Bioinformatics.

[21] Rosen B.D., Bickhart D.M., Schnabel R.D., Koren S., Elsik C.G., Tseng E., Rowan T.N., Low W.Y., Zimin A., Couldrey C. (2020). De Novo Assembly of the Cattle Reference Genome with Single-Molecule Sequencing. GigaScience.

[22] Anders S., Reyes A., Huber W. (2012). Detecting Differential Usage of Exons from RNA-Seq Data. Genome Res..

[23] Danecek P., Bonfield J.K., Liddle J., Marshall J., Ohan V., Pollard M.O., Whitwham A., Keane T., McCarthy S.A., Davies R.M. (2021). Twelve Years of SAMtools and BCFtools. GigaScience.

[24] Kolberg L., Raudvere U., Kuzmin I., Adler P., Vilo J., Peterson H. (2023). g:Profiler—Interoperable Web Service for Functional Enrichment Analysis and Gene Identifier Mapping (2023 Update). Nucleic Acids Res..

[25] Roof D.J., Hayes A., Adamian M., Chishti A.H., Li T. (1997). Molecular Characterization of AbLIM, a Novel Actin-Binding and Double Zinc Finger Protein. J. Cell Biol..

[26] Gitai Z., Yu T.W., Lundquist E.A., Tessier-Lavigne M., Bargmann C.I. (2003). The Netrin Receptor UNC-40/DCC Stimulates Axon Attraction and Outgrowth Through Enabled and, in Parallel, Rac and UNC-115/AbLIM. Neuron.

[27] Ohsawa N., Koebis M., Mitsuhashi H., Nishino I., Ishiura S. (2015). ABLIM1 Splicing Is Abnormal in Skeletal Muscle of Patients with DM1 and Regulated by MBNL, CELF and PTBP1. Genes Cells.

[28] Gandal M.J., Haney J.R., Parikshak N.N., Leppa V., Ramaswami G., Hartl C., Schork A.J., Appadurai V., Buil A., Werge T.M. (2018). Shared Molecular Neuropathology Across Major Psychiatric Disorders Parallels Polygenic Overlap. Science.

[29] Flores C., Manitt C., Rodaros D., Thompson K.M., Rajabi H., Luk K.C., Tritsch N.X., Sadikot A.F., Stewart J., Kennedy T.E. (2005). Netrin Receptor Deficient Mice Exhibit Functional Reorganization of Dopaminergic Systems and Do Not Sensitize to Amphetamine. Mol. Psychiatry.

[30] Hoops D., Yee Y., Hammill C., Wong S., Manitt C., Bedell B.J., Cahill L., Lerch J.P., Flores C., Sled J.G. (2024). Disproportionate Neuroanatomical Effects of DCC Haploinsufficiency in Adolescence Compared with Adulthood: Links to Dopamine, Connectivity, Covariance, and Gene Expression Brain Maps in Mice. J. Psychiatry Neurosci..

[31] Camerota M., Lester B.M., McGowan E.C., Carter B.S., Check J., Dansereau L.M., DellaGrotta S.A., Helderman J.B., Hofheimer J.A., Loncar C.M. (2024). Contributions of Prenatal Risk Factors and Neonatal Epigenetics to Cognitive Outcome in Children Born Very Preterm. Dev. Psychol..

[32] Golanska E., Sieruta M., Gresner S.M., Pfeffer A., Chodakowska-Zebrowska M., Sobow T.M., Klich I., Mossakowska M., Szybinska A., Barcikowska M. (2013). APBB2 Genetic Polymorphisms Are Associated with Severe Cognitive Impairment in Centenarians. Exp. Gerontol..

[33] Vandenbriele C., Kauskot A., Vandersmissen I., Criel M., Geenens R., Craps S., Luttun A., Janssens S., Hoylaerts M.F., Verhamme P. (2015). Platelet Endothelial Aggregation Receptor-1: A Novel Modifier of Neoangiogenesis. Cardiovasc. Res..

[34] Thomas G., Aslan J.E., Thomas L., Shinde P., Shinde U., Simmen T. (2017). Caught in the Act—Protein Adaptation and the Expanding Roles of the PACS Proteins in Tissue Homeostasis and Disease. J. Cell Sci..

[35] Zhang H., Gao K., Wang S., Zhang Y.H., Yang Z.X., Wu Y., Jiang Y.W. (2024). PACS Gene Family-Related Neurological Diseases: Limited Genotypes and Diverse Phenotypes. World J. Pediatr..

[36] Hussain T., Tan B., Ren W., Rahu N., Kalhoro D.H., Yin Y. (2017). Exploring Polyamines: Functions in Embryo/Fetal Development. Anim. Nutr..

[37] Prokop J.W., Bupp C.P., Frisch A., Bilinovich S.M., Campbell D.B., Vogt D., Schultz C.R., Uhl K.L., VanSickle E., Rajasekaran S. (2021). Emerging Role of ODC1 in Neurodevelopmental Disorders and Brain Development. Genes.

[38] Jeanne M., Demory H., Moutal A., Vuillaume M.L., Blesson S., Thépault R.A., Marouillat S., Halewa J., Maas S.M., Motazacker M.M. (2021). Missense Variants in DPYSL5 Cause a Neurodevelopmental Disorder with Corpus Callosum Agenesis and Cerebellar Abnormalities. Am. J. Hum. Genet..

[39] Feuer K.L., Peng X., Yovo C.K., Avramopoulos D. (2023). DPYSL2/CRMP2 Isoform B Knockout in Human iPSC-Derived Glutamatergic Neurons Confirms Its Role in mTOR Signaling and Neurodevelopmental Disorders. Mol. Psychiatry.

[40] Desprez F., Ung D.C., Vourc’h P., Jeanne M., Laumonnier F. (2023). Contribution of the Dihydropyrimidinase-Like Proteins Family in Synaptic Physiology and in Neurodevelopmental Disorders. Front. Neurosci..

[41] Goudreault M., D’Ambrosio L.M., Kean M.J., Mullin M.J., Larsen B.G., Sanchez A., Chaudhry S., Chen G.I., Sicheri F., Nesvizhskii A.I. (2009). A PP2A Phosphatase High Density Interaction Network Identifies a Novel Striatin-Interacting Phosphatase and Kinase Complex Linked to the Cerebral Cavernous Malformation 3 (CCM3) Protein. Mol. Cell. Proteom..

[42] Bazzi H., Soroka E., Alcorn H.L., Anderson K.V. (2017). STRIP1, a Core Component of STRIPAK Complexes, Is Essential for Normal Mesoderm Migration in the Mouse Embryo. Proc. Natl. Acad. Sci. USA.

[43] Wang J., Manick B., Renelt M., Suin J., Hansen L., Person A., Kalabokis V., Wu G. (2019). VSTM4 Is a Novel Negative Regulator of T Cell Activation. J. Immunol..

[44] Lesseur C., Kaur K., Kelly S.D., Hermetz K., Williams R., Hao K., Marsit C.J., Caudle W.M., Chen J. (2023). Effects of Prenatal Pesticide Exposure on the Fetal Brain and Placenta Transcriptomes in a Rodent Model. Toxicology.

[45] Li Y., Schrodi S., Rowland C., Tacey K., Catanese J., Grupe A. (2006). Genetic Evidence for Ubiquitin-Specific Proteases USP24 and USP40 as Candidate Genes for Late-Onset Parkinson Disease. Hum. Mutat..

[46] Berthon A., Faucz F.R., Espiard S., Drougat L., Bertherat J., Stratakis C.A. (2017). Age-Dependent Effects of Armc5 Haploinsufficiency on Adrenocortical Function. Hum. Mol. Genet..

[47] Potkin S.G., Turner J.A., Fallon J.A., Lakatos A., Keator D.B., Guffanti G., Macciardi F. (2009). Gene Discovery Through Imaging Genetics: Identification of Two Novel Genes Associated with Schizophrenia. Mol. Psychiatry.

[48] Vissers L.E.L.M., Kalvakuri S., de Boer E., Geuer S., Oud M., van Outersterp I., Kwint M., Witmond M., Kersten S., Polla D.L. (2020). De Novo Variants in CNOT1, a Central Component of the CCR4-NOT Complex Involved in Gene Expression and RNA and Protein Stability, Cause Neurodevelopmental Delay. Am. J. Hum. Genet..

[49] Guo W., Cai Y., Zhang H., Yang Y., Yang G., Wang X., Zhao J., Lin J., Zhu J., Li W. (2017). Association of ARHGAP18 Polymorphisms with Schizophrenia in the Chinese-Han Population. PLoS ONE.

[50] Lombardo A.T., Mitchell C.A., Zaman R., McDermitt D.J., Bretscher A. (2024). ARHGAP18-Ezrin Functions as an Autoregulatory Module for RhoA in the Assembly of Distinct Actin-Based Structures. eLife.

[51] Streit L., Brunaud L., Vitale N., Ory S., Gasman S. (2020). Hormones Secretion and Rho GTPases in Neuroendocrine Tumors. Cancers.

[52] Hodes A., Lichtstein D. (2014). Natriuretic Hormones in Brain Function. Front. Endocrinol..

[53] Perez-Ternero C., Pallier P.N., Tremoleda J.L., Delogu A., Fernandes C., Michael-Titus A.T., Hobbs A.J. (2022). C-type natriuretic peptide preserves central neurological function by maintaining blood-brain barrier integrity. Front. Mol. Neurosci..

[54] Doycheva D., Xu N., Kaur H., Malaguit J., McBride D.W., Tang J., Zhang J.H. (2019). Adenoviral TMBIM6 Vector Attenuates ER-Stress-Induced Apoptosis in a Neonatal Hypoxic-Ischemic Rat Model. Dis. Model. Mech..

[55] Zhang Y., Xue L., Xu H., Liang W., Wu Q., Zhang Q., Zhou X., Liu B. (2020). Global Analysis of Alternative Splicing Difference in Peripheral Immune Organs Between Tongcheng Pigs and Large White Pigs Artificially Infected with PRRSV In Vivo. BioMed Res. Int..

[56] Aubol B.E., Fattet L., Adams J.A. (2021). A Conserved Sequence Motif Bridges Two Protein Kinases for Enhanced Phosphorylation and Nuclear Function of a Splicing Factor. FEBS J..

[57] Erkelenz S., Grzonka M., Papadakis A., Schaal H., Hoeijmakers J.H.J., Gyenis Á. (2024). Rbm3 Deficiency Leads to Transcriptome-Wide Splicing Alterations. RNA Biol..

[58] Coci E.G., Codutti L., Fink C., Bartsch S., Grüning G., Lücke T., Kurth I., Riedel J. (2017). Novel Homozygous Missense Mutation in ALDH7A1 Causes Neonatal Pyridoxine Dependent Epilepsy. Mol. Cell. Probes.

[59] Soriano-Herrador C., Ubeda-Banon I., Villanueva-Anguita P., Saiz-Sanchez D., Astillero-Lopez V., Martinez-Marcos A., Flores-Cuadrado A. (2025). The Chaperones HSP90AA1, HSP90AB1 and BAG3 Are Specifically Distributed Among Human Hippocampal Subfields During Different Alzheimer’s Disease Stages. Neurobiol. Dis..

[60] Wang Z., Yan X., Zhao C. (2017). Dynamical Differential Networks and Modules Inferring Disrupted Genes Associated with the Progression of Alzheimer’s Disease. Exp. Ther. Med..

[61] Broeks M.H., Shamseldin H.E., Alhashem A., Hashem M., Abdulwahab F., Alshedi T., Alobaid I., Zwartkruis F., Westland D., Fuchs S. (2019). MDH1 Deficiency Is a Metabolic Disorder of the Malate-Aspartate Shuttle Associated with Early Onset Severe Encephalopathy. Hum. Genet..

[62] Fregeau B., Kim B.J., Hernández-García A., Jordan V.K., Cho M.T., Schnur R.E., Monaghan K.G., Juusola J., Rosenfeld J.A., Bhoj E. (2016). De Novo Mutations of RERE Cause a Genetic Syndrome with Features That Overlap Those Associated with Proximal 1p36 Deletions. Am. J. Hum. Genet..

[63] Katsuki A., Kakeda S., Watanabe K., Igata R., Otsuka Y., Kishi T., Nguyen L., Ueda I., Iwata N., Korogi Y. (2019). A Single-Nucleotide Polymorphism Influences Brain Morphology in Drug-Naïve Patients with Major Depressive Disorder. Neuropsychiatr. Dis. Treat..

[64] Li Q., Li W., Hu K., Wang Y., Li Y., Xu J. (2024). A De Novo Variant in RERE Causes Autistic Behavior by Disrupting Related Genes and Signaling Pathway. Clin. Genet..

[65] Autism Spectrum Disorders Working Group of The Psychiatric Genomics Consortium (2017). Meta-Analysis of GWAS of Over 16,000 Individuals with Autism Spectrum Disorder Highlights a Novel Locus at 10q24.32 and a Significant Overlap with Schizophrenia. Mol. Autism.

[66] Núñez-Ríos D.L., Nagamatsu S.T., Martínez-Magaña J.J., Montalvo-Ortiz J.L., Traumatic Stress Brain Research Group (2025). Integrated Gene and Isoform-Level Transcriptomic Analysis of Adverse Childhood Experiences in the Human Prefrontal Cortex. Yale J. Biol. Med..

[67] Wojcik S.M., Tantra M., Stepniak B., Man K.N., Müller-Ribbe K., Begemann M., Ju A., Papiol S., Ronnenberg A., Gurvich A. (2013). Genetic Markers of a Munc13 Protein Family Member, BAIAP3, Are Gender Specifically Associated with Anxiety and Benzodiazepine Abuse in Mice and Humans. Mol. Med..

[68] Turi da Fonte Dias A.R., Hoffmann I. (2025). The E3 Ubiquitin Ligase SPRYD3-MYCBP2(PAM) Regulates Mitotic Cell Fate and Ubiquitination of USP11 to Control Spindle Assembly. J. Biol. Chem..

[69] Dehkordi S.K., Sajedi S., Heshmat A., Orr M.E., Zare H. (2025). Identification of Markers for Neurescence Through Transcriptomic Profiling of Postmortem Human Brains. npj Aging.

[70] Jo C., Gundemir S., Pritchard S., Jin Y.N., Rahman I., Johnson G.V. (2014). Nrf2 Reduces Levels of Phosphorylated Tau Protein by Inducing Autophagy Adaptor Protein NDP52. Nat. Commun..

[71] Frank M., Schaeren-Wiemers N., Schneider R., Schwab M.E. (1999). Developmental Expression Pattern of the Myelin Proteolipid MAL Indicates Different Functions of MAL for Immature Schwann Cells and in a Late Step of CNS Myelinogenesis. J. Neurochem..

[72] Dal Molin E.A., Kaster M.P., Nascimento J.M. (2026). Glucocorticoid Signaling in PSC-Derived Neural Systems to Elucidate Mechanisms of Stress-Induced Psychiatric Vulnerability. Mol. Neurobiol..

[73] Gismondi A., Caldarola S., Lisi G., Juli G., Chellini L., Iadevaia V., Proud C.G., Loreni F. (2014). Ribosomal Stress Activates eEF2K–eEF2 Pathway Causing Translation Elongation Inhibition and Recruitment of Terminal Oligopyrimidine (TOP) mRNAs on Polysomes. Nucleic Acids Res..

[74] Pleiss J.A., Whitworth G.B., Bergkessel M., Guthrie C. (2007). Rapid, Transcript-Specific Changes in Splicing in Response to Environmental Stress. Mol. Cell.

[75] Xue S., Barna M. (2012). Specialized Ribosomes: A New Frontier in Gene Regulation and Organismal Biology. Nat. Rev. Mol. Cell Biol..

[76] Shi Z., Fujii K., Kovary K.M., Genuth N.R., Röst H.L., Teruel M.N., Barna M. (2017). Heterogeneous Ribosomes Preferentially Translate Distinct Subpools of mRNAs Genome-Wide. Mol. Cell.

[77] Baker E.C., Earnhardt A.L., Cilkiz K.Z., Collins H.C., Littlejohn B.P., Cardoso R.C., Ghaffari N., Long C.R., Riggs P.K., Randel R.D. (2022). DNA Methylation Patterns and Gene Expression from Amygdala Tissue of Mature Brahman Cows Exposed to Prenatal Stress. Front. Genet..

