# Supplementary material for: Differential Exon Usage in Expression Profiles from Stress Axis Tissues of Brahman Cows Subjected to Prenatal Stress
Source: Genes (Basel). 2026 Sep 29;17(10):1208. doi: 10.3390/genes17101208 (PMC13704069; doi:10.3390/genes17101208)
Supplement: Supplementary file 1 [file genes-17-01208-s001.zip › genes-4534585-supplementary.pdf]

Supplementary Table S1. Functional enrichment analysis of genes with differentially used exons by tissue.

Source	Name	Adjusted *P*-value	Genes overlapping (n)	Gene IDs
Adrenal Cortex				
REAC	Caspase activation via Dependence Receptors in the absence of ligand	0.0499	1	*DCC*
Hypothalamic paraventricular nucleus				
GO:CC	cytosol	0.0003	15	*RPS6, RPL38, PPIA, RPS17, CUEDC2, CDKN2D, DPYSL2, RPL13, PRMT1, MDH1, BAIAP3, ALDH7A1, CALCOCO2, HSP90AB1, PRKAR1A*
GO:CC	cytosolic ribosome	0.0126	4	*RPS6, RPL38, RPS17, RPL13*
REAC	GTP hydrolysis and joining of the 60S ribosomal subunit	0.0037	4	*RPS6, RPL38, RPS17, RPL13*
REAC	SRP-dependent cotranslational protein targeting to membrane	0.0048	4	*RPS6, RPL38, RPS17, RPL13*
REAC	Nonsense Mediated Decay (NMD) independent of the Exon Junction Complex (EJC)	0.0050	4	*RPS6, RPL38, RPS17, RPL13*
REAC	Formation of a pool of free 40S subunits	0.0063	4	*RPS6, RPL38, RPS17, RPL13*
REAC	L13a-mediated translational silencing of Ceruloplasmin expression	0.0092	4	*RPS6, RPL38, RPS17, RPL13*
REAC	Nonsense Mediated Decay (NMD) enhanced by the Exon Junction Complex (EJC)	0.0095	4	*RPS6, RPL38, RPS17, RPL13*
REAC	Nonsense-Mediated Decay (NMD)	0.0095	4	*RPS6, RPL38, RPS17, RPL13*
REAC	Cap-dependent Translation Initiation	0.0120	4	*RPS6, RPL38, RPS17, RPL13*
REAC	Eukaryotic Translation Initiation	0.0120	4	*RPS6, RPL38, RPS17, RPL13*
REAC	Metabolism of RNA	0.0481	6	*HNRNPA2B1, RPS6, RPL38, RPS17, SNRPN, RPL13*
